# Correlation between blood pressure variability and prognosis of endovascular therapy in ischemic stroke due to middle cerebral artery occlusion

**DOI:** 10.3389/fneur.2026.1723520

**Published:** 2026-02-20

**Authors:** Hao Tao, Yi Li, Huan Liu, Xiang Fan, Meng-Yu Zhong, Jian-Hong Wang, Shu Yang, Neng-Wei Yu, Bing-Hu Li

**Affiliations:** 1 School of Medicine, University of Electronic Science and Technology of China, Chengdu, China; 2 Department of Neurology, Sichuan Provincial People’s Hospital, Chengdu, China; 3 School of Clinical Medicine, Southwest Medical University, Luzhou, China

**Keywords:** blood pressure variability, endovascular treatment, mechanical thrombectomy, outcome, stroke

## Abstract

**Purpose:**

To evaluate the impact of post-endovascular therapy (EVT) systolic, diastolic, and pulse pressure (PP) variability on 3 month functional outcomes in patients with middle cerebral artery occlusion.

**Methods:**

Patients were classified as having good (mRS 0–2) or poor (mRS 3–6) 90 day outcomes. We compared 48 h postoperative BP parameters and BPV indices (SD, CV, and ARV). Multivariable logistic regression adjusted for age, baseline NIHSS, hypertension, atrial fibrillation, and pre-stroke mRS was used to examine associations between BPV and outcome.

**Results:**

The average real variability of systolic blood pressure (SBPARV: 10.08 vs. 7.89, *p* < 0.001), diastolic blood pressure (DBPARV: 8.42 vs. 7.87, *p* = 0.006), and PP (PPARV: 11.00 vs. 8.26, *p* < 0.001) were significantly higher in the poor prognosis group than in the good prognosis group. Multivariable logistic regression showed that SBPARV (OR = 2.619, 95% CI 1.606–4.271; *p* < 0.001) and PPARV (PPARV: 11.00 vs. 8.26, *p* < 0.001) were independently associated with poor prognosis at 3 months.

**Conclusion:**

In this retrospective cohort, higher postoperative SBP ARV and PP ARV were independently associated with poor 90 day functional outcome after EVT; however, given the small sample size and observational design, the predictive value of BPV parameters is likely limited. BPV may be an epiphenomenon of more severe stroke rather than a causal determinant or clinically actionable target.

## Introduction

Acute ischemic stroke (AIS) is a significant cause of mortality and disability globally (1). Etiologically, large vessel occlusion (LVO), especially middle cerebral artery (MCA) occlusion is the major cause of AIS. EVT has been established as the most effective treatment for improving 3 month functional prognosis of the patients in previous randomized controlled trials (2, 3). However, in clinical practice, it has been observed that some patients achieved good flow recanalization (mTICI grade 2b-3) after EVT promptly but still experienced poor prognosis, which is known as “futile recanalization” (FR). Blood pressure variability (BPV) is an umbrella term that encompasses BP fluctuations over different time scales, ranging from beat-to-beat (very short-term), to 24 h ambulatory BPV (short-term), to day-to-day home BPV (mid-term), and visit-to-visit BPV over clinic visits (long-term). In the present study, we focused on short-term in-hospital postoperative BPV over the first 48 h, quantified using time-domain indices (SD, CV, and ARV) derived from intermittent cuff measurements (4). Evidence indicates even after adjusting for the effects of elevated blood pressure, BPV is still independently associated with increased risk of cardiovascular events (5). In addition, PP, another parameter of blood pressure reflecting large artery stiffness, is associated with recurrent stroke and short-term decline in neurological function (6–8). Although prior studies have linked early post-procedural BPV to outcomes, evidence regarding the prognostic relevance of PP variability remains limited. Therefore, we evaluated blood pressure dynamics during the first 48 h after EVT and, within a unified analytic framework, separately examined BPV derived from systolic, diastolic, and PP (with ARV as the primary BPV metric) to clarify their respective associations with 90 day functional outcome. The aim of this study was to evaluate the combined effect of systolic blood pressure (SBP), diastolic blood pressure (DBP), and PP variability on prognosis of EVT in AIS due to MCA occlusion.

## Method

### Population

This is a single-center retrospective cohort study that included patients with AIS with MCA occlusion who underwent EVT from January 2021 to December 2023 in a tertiary hospital.

### Inclusion and exclusion criteria

**Figure 1:**
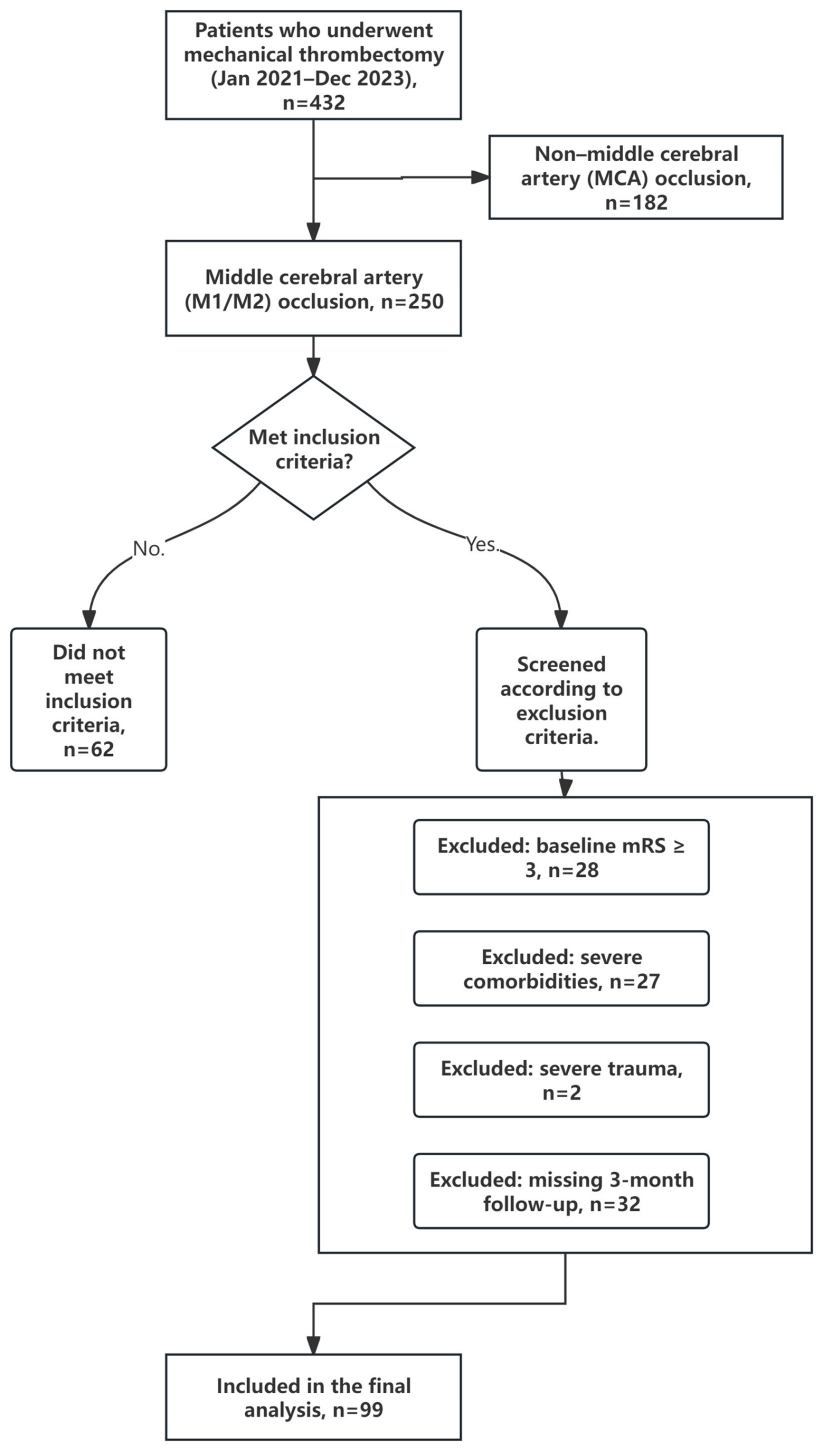
Patient selection flow diagram.

The inclusion criteria for this study are as follows: (1) The occlusion of the M1 or M2 segment of the MCA must be confirmed by CT angiography (CTA) or digital subtraction angiography (DSA); (2) mTICI 2b-3 was confirmed on postoperative DSA; (3) Postoperative blood pressure was measured using intermittent noninvasive oscillometric cuff measurements for at least 48 h. The exclusion criteria include: (1) baseline modified Rankin Scale (mRS) score ≥3 before the onset of the index AIS; (2) presence of any of the following severe comorbidities: advanced malignant tumor (expected survival <6 months); end-stage cardiac insufficiency (New York Heart Association Class IV), hepatic failure (Child-Pugh Class C), renal failure (dialysis-dependent), or respiratory failure (oxygen therapy-dependent); index AIS causing severe trauma (including but not limited to skull fractures, and comminuted limb, trunk, or spinal cord injuries); (3) loss of 3 month postoperative follow-up data (see Figure 1).

### Data collection

The primary data set encompassed demographic information, including gender, age, smoking history, a history of diabetes mellitus (in accordance with the World Health Organization’s diagnostic criteria for diabetes mellitus), a history of hypertension (SBP ≥140 mmHg or DBP ≥90 mmHg), a history of prior stroke, pre-stroke mRS [Pre-stroke mRS was highly skewed (0 in 87/99 patients); therefore, it was dichotomized as 0 vs. 1-2 for regression analyses], preadmission anticoagulation (warfarin, rivaroxaban, etc.) or antiplatelet medications (e.g., aspirin, clopidogrel, etc.). Baseline NIHSS was independently evaluated by two board-certified neurologists. The procedure-related information included: times from onset to puncture, times from puncture to recanalization, times from onset to recanalization, treatment strategy (thrombolysis, thrombolysis + thrombectomy), and method of thrombectomy (aspiration, retrieval stent, or both). Pre-procedure baseline platelet levels and baseline blood glucose levels and post-procedure glycosylated hemoglobin (HbA1c), triglycerides, total cholesterol, low-density lipoprotein (LDL), and other relevant parameters were also collected.

#### Blood pressure measurement

Postoperative blood pressure was measured using an automated non-invasive oscillometric cuff device (Omron HBP-1300). BP measurements were initiated upon arrival to the ward after EVT and continued for the first 48 h. According to routine clinical practice, measurements were typically obtained every 1 h in the ICU and every 2 h on the general ward; in the present cohort, 42 patients were monitored at 1 h intervals and 57 patients at 2 h intervals during the 48 h postoperative window. All valid BP readings within the 48 h window were used to derive mean BP and BPV indices. Patients with fewer than 20 BP readings during the 48 h period were excluded from BPV analyses, and missing values were not imputed. Implausible readings were excluded if they were clearly outside physiologic ranges and showed excessive deviation from adjacent measurements (e.g., SBP < 50 or >250 mmHg; DBP < 30 or >150 mmHg). When repeated measurements were recorded at the same time point, the last successful reading was used for analysis.

The following metrics were employed to quantify BPV in this study: standard deviation (SD) = $\sqrt{(1/(N-1))\sum_{i=1}^{N}{\left(x_{i}-\bar{x}\right)}^{2}}$ ; coefficient of variation (CV) = $(\mathrm{SD}/\bar{x})\times 100\%$ ; average real variability (ARV) = $(1/(N-1))\sum_{i=1}^{N-1}|x_{i+1}-x_{i}|$ (ARV is calculated based on adjacent measurements arranged in chronological order).

### Outcomes

The primary functional outcome was assessed using the mRS at 3 months post-procedure and mRS score of ≤2 was defined as a good prognosis.

### Statistical analysis

The analysis was conducted using SPSS 26.0 statistical software, and a two-sided *p*-value less than 0.05 was considered statistically significant. Data that conformed to a normal distribution were expressed as the mean ± standard deviation (*M* ± SD), and an independent samples *t*-test was used. Non-normally distributed data were expressed as a median and interquartile range [*M* (Q25, Q75)] and were tested using the Mann-Whitney *U* test. The *χ*^2^ test or Fisher exact test was used to compare categorical variables. Independent predictors were screened using binary logistic regression analysis with 3 month functional prognosis (mRS 0–2 vs. 3–6) as the dependent variable. Because these BPV indices are mathematically correlated, only one BPV index was included in a given model; ARV (a method that accounts for the temporal sequence of blood pressure measurements and thus provides a superior quantification of BPV compared to SD and CV) was selected as the primary BPV metric. SBP-, DBP-, and PP-related BPV metrics were evaluated in separate models to reduce collinearity. Models were adjusted for clinically relevant covariates and those associated with outcome in univariable analyses. Results are presented as odds ratios (ORs) with 95% confidence intervals (CIs).

## Results

### Comparison of baseline data between the two groups

A total of 99 patients were enrolled in this study, comprising 55 (55.6%) males and 44 (44.4%) females, with a mean (SD) age of 67.6 ± 12.8 years. The clinical characteristics were as follows: diabetes mellitus in 27 (27.3%), hypertension in 57 (57.6%), and atrial fibrillation in 42 (42.4%). The median baseline NIHSS score was 13 (interquartile range: 9–17), and 71 (71.7%) achieved complete recanalization (mTICI grade 3). Based on 3 month mRS, all patients were classified into good-prognosis (mRS 0–2, *n* = 34) and poor-prognosis (mRS 3–6, *n* = 65) groups. Patients in the good-prognosis group (58.3 ± 12.6 years) were younger compared to those in the poor-prognosis group (72.4 ± 9.9 years) (*p* < 0.001). Furthermore, the good-prognosis group showed a lower prevalence of hypertension (41.2% vs. 66.2%, *p* = 0.017) and atrial fibrillation (26.5% vs. 50.8%, *p* = 0.020) compared to the poor-prognosis group (Table 1).

**Table 1:** Comparison of baseline data between the two groups.

Characteristics	Overall, *n* = 99	Good-prognosis (mRS 0–2), *n* = 34	Poor-prognosis (mRS 3–6), *n* = 65	*p*-value
Sex, males, *n* (%)	55, (55.6)	19 (55.9)	36 (55.4)	0.962
Age, years, mean ± SD	67.56 ± 12.79	58.29 ± 12.60	72.40 ± 9.95	<0.001
Smoking history, *n* (%)	40 (40.4)	16 (47.1)	24 (36.9)	0.329
Diabetes mellitus, *n* (%)	27 (27.3)	8 (23.5)	19 (29.2)	0.545
Hypertension, *n* (%)	57 (57.6)	14 (41.2)	43 (66.2)	0.017
Atrial fibrillation, *n* (%)	42 (42.4)	9 (26.5)	33 (50.8)	0.020
Pre-stroke mRS (1-2), *n* (%)	12 (12.1)	3 (8.8)	9 (13.8)	0.537*
History of anticoagulant/antiplatelet medication use, *n* (%)	19 (19.2)	6 (17.6)	13 (20)	0.778
Baseline NIHSS score, median (IQR)	13 (9, 17)	11 (8, 17)	13 (11, 17)	0.050
Time from onset to puncture, min, median (IQR)	368 (234, 599)	372 (232, 720)	362 (238, 522)	0.648
Time from puncture to recanalization, min, median (IQR)	46 (31, 68)	51.5 (32, 63)	43 (31, 68)	0.682
Time from onset to recanalization, min, median (IQR)	407 (291, 643)	411 (291, 798)	407 (311, 600)	0.842
mTICI **= 3**, *n* (%)	71 (71.7)	26 (76.5)	45 (69.2)	0.577
Baseline platelet count, ×10^9^/L, mean ± SD	181.30 ± 62.21	189.50 ± 55.16	177.26 ± 65.42	0.365
Baseline blood glucose level, mmol/L, median (IQR)	7.46 (5.84, 9.83)	6.24 (5.16, 8.86)	7.61 (6.26, 10.22)	0.080
HbA1c, %, median (IQR)	5.89 (5.55, 6.40)	5.72 (5.53, 6.11)	5.91 (5.55, 6.74)	0.303
TC, mmol/L, mean ± SD	4.55 ± 1.13	4.79 ± 1.05	4.42 ± 1.16	0.124
TG, mmol/L, median (IQR)	1.13 (0.77, 1.53)	1.35 (0.84, 1.83)	1.10 (0.85, 1.43)	0.020
LDL, mmol/L, mean ± SD	2.72 ± 1.03	2.94 ± 0.94	2.61 ± 1.06	0.131

HbA1c, Glycated hemoglobin A1c; TC, Total cholesterol; TG, Triglycerides; LDL, Low-density lipoprotein; NIHSS, National Institutes of Health Stroke Scale. *Fisher’s exact test.

### Comparison of SBP and its variability between the two groups

Based on SBP, related parameters including maximum SBP (SBPmax), minimum SBP (SBPmin), mean SBP (SBPmean), standard deviation (SBPSD), coefficient of variation (SBPCV), and average real variability (SBPARV) were compared between good-prognosis and poor-prognosis group. As shown in Table 2, there were significant differences in SBPmax, SBPSD, SBPCV, SBPARV, SBPmean between the two groups (all *p* < 0.05). There were no significant differences in SBPmin between the two groups (*p* = 0.845). SBP variability indices (including SBPSD, SBPCV, and SBPARV) in the poor-prognosis group were notably elevated in comparison to those observed in the good-prognosis group (all *p* < 0.001), indicating that increased systolic BPV is associated with a poor prognosis (Table 2).

**Table 2:** Comparison of SBP and its variability between the two groups.

SBP metric	Overall, *n* = 99	Good-prognosis (mRS 0–2), *n* = 34	Poor-prognosis (mRS 3–6), *n* = 65	*p*-value
SBPmax, mmHg, median (IQR)	149 (137, 164)	138 (130, 151)	156 (143, 168)	<0.001
SBPmin, mmHg, median (IQR)	98 (91, 105)	100 (92, 101)	98 (91, 109)	0.845
SBPmean, mmHg, median (IQR)	123 (113, 130)	118 (111, 126)	125 (115, 132)	0.006
SBPSD, mean ± SD	12.20 ± 3.37	10.28 ± 3.05	13.21 ± 3.10	<0.001
SBPCV, %, median (IQR)	10.02 (7.86, 11.60)	8.05 (6.82, 10.44)	10.45 (8.63, 12.08)	<0.001
SBPARV, median, (IQR)	9.32 (7.94, 10.69)	7.89 (6.53, 9.44)	10.08 (8.69, 11.69)	<0.001

SBP, Systolic blood pressure; SBPmax, Maximum systolic blood pressure; SBPmin, Minimum SBP; SBPmean, Mean SBP; SBPSD, Standard deviation of SBP; SBPCV, Coefficient of variation of SBP; SBPARV, Average real variability of SBP.

### Comparison of DBP and its variability between the two groups

Based on DBP, the following parameters were compared between the good-prognosis and poor-prognosis groups: maximum DBP (DBPmax), minimum DBP (DBPmin), mean DBP (DBPmean), standard deviation (DBPSD), coefficient of variation (DBPCV), and average real variability (DBPARV). As shown in Table 3, significant differences were observed in DBPmax, DBPSD, DBPCV and DBPARV between the two groups (all *p* < 0.05), whereas no significant differences were found in DBPmin or DBPmean (*p* = 0.721 and 0.505, respectively). Further analysis demonstrated that DBPV indices—including DBPSD, DBPCV, and DBPARV—were significantly elevated in the poor-prognosis group compared to the good-prognosis group (all *p* < 0.05), suggesting that increased DBP variability is similarly associated with unfavorable outcomes (Table 3).

**Table 3:** Comparison of DBP and its variability between the two groups.

DBP metric	Overall, *n* = 99	Good-prognosis (mRS 0–2), *n* = 34	Poor-prognosis (mRS 3–6), *n* = 65	*p*-value
DBPmax, mmHg, mean ± SD	97.34 ± 12.99	91.97 ± 9.03	100.15 ± 13.89	0.002
DBPmin, mmHg, median (IQR)	52.00 (46.00, 60.00)	53.00 (45.75, 58.25)	52.00 (45.00, 61.00)	0.721
DBPmean, mmHg, mean ± SD	73.83 ± 9.50	72.94 ± 8.44	74.29 ± 10.05	0.505
DBPSD, mean ± SD	9.74 ± 2.22	8.66 ± 1.82	10.31 ± 2.21	<0.001
DBPCV, %, mean ± SD	13.33 ± 3.16	12.01 ± 2.82	14.02 ± 3.13	0.002
DBPARV, mmHg, median (IQR)	8.16 (7.13, 9.73)	7.87 (6.75, 9.48)	8.42 (7.57, 10.03)	0.006

DBP, Diastolic blood pressure; DBPmax, Maximum diastolic blood pressure; DBPmin, Minimum DBP; DBPmean, Mean DBP; DBPSD, Standard deviation of DBP; DBPCV, Coefficient of variation of DBP; DBPARV, Average real variability of DBP.

### Comparison of PP and its variability between the two groups

Based on PP, the following parameters were compared between the good-prognosis and poor-prognosis groups: maximum PP (PPmax), minimum PP (PPmin), mean PP (PPmean), standard deviation of PP (PPSD), coefficient of variation of PP (PPCV), average real variability of PP (PPARV). As shown in Table 4, significant differences were observed in PPmax and PPmean between the two groups (all *p* < 0.05), whereas no statistical difference was found in PPmin (*p* = 0.754). Further variability analysis revealed that PP variability indices—including PPSD and PPARV—were significantly elevated in the poor-prognosis group compared to the good-prognosis group (all *p* < 0.001), while no significant difference was observed in PPCV (*p* = 0.091), indicating that heightened PP variability correlates with unfavorable outcomes (Table 4).

**Table 4:** Comparison of PP and its variability between the two groups.

PP metric	Overall, *n* = 99	Good-prognosis (mRS 0–2), *n* = 34	Poor-prognosis (mRS 3–6), *n* = 65	*p*-value
PPmax, mmHg, median (IQR)	73.00 (62.00, 84.00)	63.00 (58.50, 73.50)	76.00 (66.50, 86.50)	<0.001
PPmin, mmHg, median (IQR)	25.00 (17.00, 31.00)	25.00 (19.75, 31.00)	26.00 (16.50, 31.00)	0.754
PPmean, mmHg, mean ± SD	49.36 ± 11.83	45.29 ± 10.78	51.49 ± 11.88	0.013
PPSD, median (IQR)	10.37 (8.58, 10.37)	9.43 (7.19, 11.17)	10.82 (9.61, 13.65)	<0.001
PPCV, %, median (IQR)	21.03 (17.59, 25.92)	19.53 (15.97, 25.11)	21.09 (18.42, 24.98)	0.091
PPARV, median (IQR)	9.62 (7.93, 11.65)	8.26 (6.94, 9.53)	11.00 (8.38, 12.24)	<0.001

PP, Pulse pressure; PPmax, Maximum PP; PPmin, Minimum PP; PPmean, Mean PP; PPSD, Standard deviation of PP; PPCV, Coefficient of variation of PP; PPARV, Average real variability of PP.

### Multivariate regression analysis

To reduce collinearity among BPV metrics, we constructed three separate multivariable logistic regression models, each including a single ARV parameter (SBPARV, DBPARV, or PPARV), while adjusting for age, baseline NIHSS score, hypertension, atrial fibrillation, and pre-stroke mRS (Although pre-stroke mRS did not show a statistically significant between-group difference in the univariable analysis, our primary endpoint was the 90 day mRS, and baseline functional status is clinically and prognostically important for post-stroke outcomes. Therefore, we included pre-stroke mRS as an *a priori* covariate in the multivariable regression models to account for potential confounding). In Model 1, higher SBPARV was independently associated with poor 90 day functional outcome (mRS 3–6) (OR = 2.619, 95% CI 1.606–4.271; *p* < 0.001). In Model 2, DBPARV was not significantly associated with outcome (OR = 1.132, 95% CI 0.889–1.440; *p* = 0.315). In Model 3, higher PPARV remained independently associated with poor outcome (OR = 1.301, 95% CI 1.054–1.605; *p* = 0.014). Age was consistently associated with poor outcome across models, whereas pre-stroke mRS, hypertension, and atrial fibrillation were not statistically significant. Baseline NIHSS score was significant in Model 1 and showed borderline significance in Models 2-3 (see Table 5).

**Table 5:** Multivariate regression analysis.

Variable	Model 1: SBPARV aOR (95% CI), *p*	Model 2: DBPARV aOR (95% CI), *p*	Model 3: PPARV aOR (95% CI), *p*
Age	1.127 (1.053–1.207), 0.001	1.117 (1.055–1.183), <0.001	1.113 (1.050–1.180), <0.001
Baseline NIHSS	1.129 (1.015–1.255), 0.025	1.081 (0.998–1.172), 0.057	1.087 (0.999–1.182), 0.052
Hypertension	0.965 (0.264–3.527), 0.957	0.512 (0.179–1.465), 0.212	0.568 (0.192–1.683), 0.307
Atrial fibrillation	0.827 (0.188–3.633), 0.802	1.266 (0.368–4.357), 0.708	1.198 (0.334–4.292), 0.782
Pre-stroke mRS (1-2 vs. 0)	1.632 (0.237–11.214), 0.619	0.902 (0.162–5.015), 0.906	0.967 (0.169–5.533), 0.970
ARV metric	2.619 (1.606–4.271), <0.001	1.132 (0.889–1.440), 0.315	1.301 (1.054–1.605), 0.014

## Discussion

In the context of AIS with LVO of MCA, EVT emerges as a pivotal intervention to achieve reperfusion in a timely manner. The safety and efficacy of EVT have been validated within a 6 h timeframe (3), with ongoing expansion of this window through advancements in research (9). However, the development of FR has led to a situation in which nearly half of the patients are unable to benefit from EVT (10). Consequently, the identification of predictive and interventional factors associated with FR occurrence is a primary focus of current research.

Postoperative BP is a key modifiable factor after EVT and has been reported to influence outcomes (11). In the present study, we found that postoperative blood pressure was higher in the poor prognosis group than in the good-prognosis group. Although elevated postoperative blood pressure correlates with poor prognosis, aggressive blood pressure reduction post-EVT may paradoxically exacerbate neurological impairment by inducing hypoperfusion in residual ischemic areas (12, 13). Furthermore, intensive blood pressure control (target SBP 100–129 mmHg) compared to conventional blood pressure control (target SBP 130–185 mmHg) did not demonstrate a significant difference in the risk of symptomatic intracranial hemorrhage (*p* = 0.84), and lower blood pressure did not reduce the probability of transformed hemorrhage (14). Another study also demonstrated that intensive blood pressure control (target SBP < 140 mmHg) was associated with poorer functional independence (15). Suggesting that overly stringent blood pressure control may not be warranted in patients with AIS underwent successful EVT. The choice of intensive blood pressure lowering and the absence of intensive blood pressure lowering after EVT appear to be associated with adverse clinical events. The lack of consensus on standardized target values, coupled with individual differences, complicates the development of guidelines for postoperative blood pressure management.

In the present study, higher SBP ARV and PPARV, but not DBP ARV, were independently associated with poor prognosis. The between-group difference in SBPARV remained significant after adjusting for the effects of age, hypertension, atrial fibrillation, baseline NIHSS score, pre-stroke mRS. Prior studies have also suggested prognostic relevance of BPV; however, the existing literature spans heterogeneous disease populations and BP monitoring approaches. For example, BPV has been evaluated in non-stroke populations. A retrospective study on CKD and BPV revealed that SBPBPV was increased in patients with CKD compared to hypertensive patients without CKD, and worsened progressively as the stage of CKD progressed (16). In addition, elevated BPV in the acute versus subacute phase (from 72 h of onset to hospital discharge) in stroke patients has been associated with an increased risk of 3 month poor prognosis and subsequent cardiovascular events at discharge (17–19). A study by Huang et al. (20) found that patients with higher BPV as measured by SD and CV within the first 24 h after EVT had a significantly higher risk of postoperative sICH, and a significant lower odds of a favorable functional outcome. Similar findings have also been reported in two other studies using the same observation window (21, 22). Conversely, high-frequency BP oscillations during the hyperacute period (within 2 h) are also strongly associated with poor prognosis (23). Beyond single summary BPV metrics, Prasad et al. identified distinct temporal trajectories of post-EVT SBP variability over the first 72 h. Patients classified into a high-BPV trajectory exhibited higher SBP variability across the early post-EVT period and had an increased risk of poor 90 day functional outcome compared with the low-variability trajectory (24). Although these studies suggest a prognostic value of BPV, they are heterogeneous with respect to patient populations, monitoring modalities, observation windows, sampling intervals, and BPV metrics; therefore, their effect estimates cannot be directly compared with those of the present study. Notably, we did not assess visit-to-visit BPV, 24 h ambulatory BPV, or beat-to-beat frequency-domain BPV. Accordingly, our findings specifically pertain to short-term in-hospital BPV within the first 48 h after EVT, derived from intermittent cuff-based measurements.

The pathophysiologic mechanisms underlying increased BPV (SBP and DBP) are not yet fully understood. Prior studies have indicated that short- to medium-term increases in BPV may be associated with increased aortic stiffness and abnormal carotid artery remodeling (25), which could partially explain the relationship between BPV and cardiovascular disease. Chronic elevation in BPV has also been linked to a reduction in structural brain volume, particularly in the hippocampus (26). With respect to the pathophysiological mechanisms underlying the impairment of high BPV after EVT, impaired cerebral autoregulation (CA) mechanisms may be one of them; CA is a protective mechanism that maintains relatively stable cerebral blood flow (CBF) in the presence of fluctuations in cerebral perfusion pressure (CPP) or arterial blood pressure (ABP) (27). Following a cerebral infarction, this mechanism is disrupted, and large BP fluctuations directly affect intracranial blood vessels and tissues, thereby exacerbating the damage (28). Concurrently, impaired autonomic regulation following a stroke may contribute to heightened BP fluctuations, thereby further exacerbating the damage. However, given the current studies’ limitations, further research is necessary to elucidate whether elevated BPV is a cause of poorer prognosis or merely a physiological symptom in critically ill patients. While a causal relationship has not been firmly established, a reduction in BP variability is considered a favorable outcome in clinical practice. Calcium channel antagonists, a class of antihypertensive medications, have demonstrated efficacy in reducing BPV and organ damage in experimental models (29). Furthermore, clinical trials have suggested that CCBs may offer enhanced benefits in reducing BPV during follow-up periods (30). However, whether CCBs confer greater clinical benefits compared to other antihypertensive agents in post-EVT patients with comorbid hypertension remains to be further investigated and validated. The pathophysiologic effects of blood pressure fluctuations may not be limited to a single parameter dimension. PP, a major indicator of blood pressure, primarily reflects the stiffness of large arteries. Its value can vary according to the state of vascular sclerosis, with narrowing or stiffening of arteries leading to increased PP (31). A meta-analysis of studies revealed a strong correlation between elevated mean PP during the initial 7 days of AIS and an unfavorable prognosis (32). Moreover, heightened PP during the acute phase has been associated with increased risk of neurological deterioration and subsequent recurrent stroke (33, 34). In the present study, we evaluated both mean PP and PPV after EVT. PPARV remained independently associated with poor outcome after multivariable adjustment, suggesting that PPV may provide complementary prognostic information beyond mean PP. However, because most prior studies have focused on absolute PP levels and evidence on PPV is limited, the clinical applicability of PPV remains uncertain. Therefore, our findings regarding PPV should be interpreted as exploratory and hypothesis-generating, and warrant confirmation in larger prospective cohorts with standardized BP measurement protocols (and higher-resolution BP data when available) to determine whether PPV is a clinically actionable target rather than a statistical correlate.

This study has several limitations. First, the single-center retrospective design and modest sample size limit statistical power. Second, although we adjusted for clinical covariates, BPV may represent an epiphenomenon of more severe stroke and physiological instability (e.g., impaired autoregulation/autonomic dysfunction, larger infarct burden, or more intensive clinical management) rather than a causal or clinically actionable predictor. Therefore, the incremental predictive value of BPV parameters in this dataset is likely limited. Third, BP sampling intervals were not fully uniform (1 h vs. 2 h measurements), and data on vasoactive/antihypertensive medication use were not available, which may have influenced BP dynamics. Larger prospective multicenter studies with standardized BP acquisition and pre-specified prediction models are needed to validate these findings.

## Conclusion

In this retrospective cohort of 99 EVT-treated patients with MCA occlusion, SBP ARV and PP ARV during the first 48 h were independently associated with poor 90 day functional outcome. However, given the modest sample size and observational design, the predictive value of BPV parameters is likely limited, and BPV may be an epiphenomenon of more severe stroke rather than a causal determinant or clinically actionable target. These findings should be considered exploratory and warrant confirmation in larger prospective cohorts with standardized BP measurement protocols.

## Data Availability

The original contributions presented in the study are included in the article/supplementary material, further inquiries can be directed to the corresponding authors.
